# *De novo* assembly and transcriptome analysis of two contrary tillering mutants to learn the mechanisms of tillers outgrowth in switchgrass (*Panicum virgatum* L.)

**DOI:** 10.3389/fpls.2015.00749

**Published:** 2015-09-16

**Authors:** Kaijie Xu, Fengli Sun, Guaiqiang Chai, Yongfeng Wang, Lili Shi, Shudong Liu, Yajun Xi

**Affiliations:** ^1^State Key Laboratory of Crop Stress Biology for Arid Areas, College of Agronomy, Northwest A&F UniversityYangling, China; ^2^Institute of Cotton Research of CAASAnyang, China; ^3^HanDanShi Agriculture Academy of SciencesHandan, China

**Keywords:** tillering, next-Generation sequencing, switchgrass, transcriptome, differentially expressed genes

## Abstract

Tillering is an important trait in monocotyledon plants. The switchgrass (*Panicum virgatum*), studied usually as a source of biomass for energy production, can produce hundreds of tillers in its lifetime. Studying the tillering of switchgrass also provides information for other monocot crops. High-tillering and low-tillering mutants were produced by ethyl methanesulfonate mutagenesis. Alteration of tillering ability resulted from different tiller buds outgrowth in the two mutants. We sequenced the tiller buds transcriptomes of high-tillering and low-tillering plants using next-generation sequencing technology, and generated 34 G data in total. In the *de novo* assembly results, 133,828 unigenes were detected with an average length of 1,238 bp, and 5,290 unigenes were differentially expressed between the two mutants, including 3,225 up-regulated genes and 2,065 down-regulated genes. Differentially expressed gene analysis with functional annotations was performed to identify candidate genes involved in tiller bud outgrowth processes using Gene Ontology classification, Cluster of Orthologous Groups of proteins, and Kyoto Encyclopedia of Genes and Genomes pathway analysis. This is the first study to explore the tillering transcriptome in two types of tillering mutants by *de novo* sequencing.

## Introduction

Tillering is well known to play an important role in determining direct yields of monocotyledons. Tillering of seedlings is controlled by photoperiod, soil moisture, light intensity, temperature, mineral nutrition, and cutting treatment, which alter bud and stem development concurrently, as they affect vigor and over-all growth. Tillering generally comprises two steps: the initiation of tiller buds and their outgrowth. At present, tillering studies are restricted to minority grains, especially rice. Some tillering genes have been cloned. *MONOCULM1* (*MOC1*), mutation for this gene leads to rice have only one main culm without tillers, play important roles in tiller meristems initiation in rice (Li et al., 2003; Sun et al., 2010b; Lin et al., 2012; Xu et al., 2012). *OsTB1* was confirmed as a negative regulator of lateral branching in rice, presumably through expression in axillary buds (Takeda et al., 2003; Choi et al., 2012; Guo et al., 2013). Many other tillering genes have been cloned, including *OsPIN1* (Xu et al., 2005), *D3* (Yan et al., 2007; Zhao et al., 2014), *HTD1/D17* (Zou et al., 2006), *D10* (Arite et al., 2007), *D14* (Arite et al., 2009; Zhou et al., 2013), *D27* (Lin et al., 2009; Waters et al., 2012), *miR156* (Wang et al., 2008; Ling and Zhang, 2012; Chen et al., 2015), *RFL* (Deshpande et al., 2015), and *MOC3/TBA1* (Lu et al., 2015; Tanaka et al., 2015). Studies on these genes have revealed the possible molecular mechanism for initiation and outgrowth of tiller buds. Although several important regulators have been identified in the control of tillering in rice, the understanding of the tillering regulatory mechanism in other crops is still limited.

Switchgrass (Poaceae, *Panicum virgatum*) is a warm-season C4 perennial grass native to the U. S. and distributed from Mexico to Canada, and it is widely recognized as an important bioenergy crop. Switchgrass is allogamous and strongly incompatible with selfing. Research on switchgrass has expanded worldwide as a result of its desirable characteristics, which include high biomass production, ability to thrive in marginal soils with minimal input of nutrients and water, and the major carbon sink provided by its large, fibrous root system (Bouton, 2007; Schmer et al., 2008; Keshwani and Cheng, 2009). Polyploid series in *P. virgatum* ranges from diploid (2x = 18) to duodecaploid (12x = 108), and it is mostly tetraploid and hexaploid (Saski et al., 2011). There are two switchgrass ecotypes: upland and lowland. Lowland ecotypes are mainly tetraploid and are found in the southern range of the species’ distribution; upland ecotypes are primarily octaploid, exist in associations, and have wide geographical distribution (Morris et al., 2011).

Switchgrass tillers are produced throughout the life of the plant. Tillering density ranges from 12 to 30/dm^2^ in sod-forming ecotypes to 20–35/dm^2^ in bunch types (Van Esbroeck et al., 2004). Tillering number can reach several hundreds and biomass yield is approximately 74.1 t/hm^2^ (El Bassam, 1996), which is controlled by photoperiod, soil moisture, light intensity, temperature, and available nutrients, in addition to pruning treatments performed to control bud and stem development. In recent years, studies on the mechanism of tillering have been performed in switchgrass (Fu et al., 2012; Wang et al., 2013; Wuddineh et al., 2014).

Various next-generation sequencing technologies have been developed, including Solexa (Bennett et al., 2005), 454 (GS-FLX; Margulies et al., 2005), and SOLiD (Graveley, 2008; Lao et al., 2009). Although sequencing of large genomes remains costly, RNA sequencing (RNA-Seq) is an attractive alternative; by analyzing only transcribed portions of the genome, non-coding and repetitive sequences that constitute much of the genome can be avoided (Rosel et al., 2011; Sorber et al., 2011; Yao et al., 2012; Garg and Jain, 2013). These new technologies enable large quantities of DNA sequence data to be collected rapidly at lower cost, and requiring lesser effort and time, than Sanger sequencing (Fox et al., 2009). These technologies also provide a platform for studying mRNA expression levels [e.g., by selecting differentially expressed genes (DEGs)] (Marioni et al., 2008). Roche 454 gives longer reads than Illumina or SOLiD but has low accuracy in long homopolymeric regions, and Illumina is more suitable than Solid for RNA-Seq analysis (Datta et al., 2010).

The transcriptome is the complete set and number of transcripts in a cell at a specific developmental stage or under a physiological condition. Thus, transcriptome analysis is essential for interpreting the functional elements of the genome and for revealing the molecular constituents of cells and tissues (Wang et al., 2009; Wei et al., 2011; Liu et al., 2012b). With increased throughput, next-generation sequencing technologies provide an opportunity to expand sequence databases of model species (Jones-Rhoades et al., 2007; Tang et al., 2009; Mangone et al., 2010; Trapnell et al., 2010) and non-model organisms (O’Neil et al., 2010; Sun et al., 2010a; Grabherr et al., 2011; Kaur et al., 2011). Roche 454 GS-FLX Titanium technology was used to produce a comprehensive description of switchgrass transcriptome that included approximately 97 million base pairs (M bp) in the tillering stage (Wang et al., 2012). Transcriptome analysis of nodes and buds from high and low tillering switchgrass inbred lines were performed using Affymetrix gene chips (Wang et al., 2013). However, the regulation of tillering in switchgrass is still minimally understood. In this study, we performed *de novo* transcriptome sequencing using the Illumina GAIIx sequencing platform for two switchgrass mutants with different abilities of tiller bud outgrowth generated by ethyl methanesulfonate (EMS) mutagenesis, with the goal of identifying genes involved in bud outgrowth.

## Materials and Methods

### Plant Materials and RNA Extraction

Seeds from a single *P. virgatum* ‘Xiji1’ (derived from ‘Alamo’, tetraploid) plant were soaked in 1.0% EMS for 4 h to induce mutation. Two mutants were screened in the field according to tiller number and were transplanted into a glasshouse at Northwest A&F University, Yangling, China. The glasshouse conditions were as follows: temperature, 25°C; illumination intensity, 1200 lx; and photoperiod, 12-h light/12-h dark. The tillering mutants were named “*ht*” (high tillering) and “*lt*” (low tillering); large numbers of propagules were obtained from each mutant by asexual reproduction. Tiller buds with a length of 3–4 cm were separated to analyze the transcriptome. Samples from two mutants were prepared twice by double-cutting.

Total RNA was extracted from these materials using Trizol reagent (Invitrogen Life Technologies, USA) according to the standard protocol. The quality and quantity of total RNA were determined by NanoDrop (Thermo Scientific, Wilmington, DE, USA) and Agilent 2100 Bioanalyzer (Agilent Technologies, Santa Clara, CA, USA).

### cDNA Library Preparation and Illumina Sequencing

cDNA from each mutant was prepared to construct a cDNA library and sequence. Eight cDNA libraries of *ht* and *lt* were prepared according to the manufacturer’s instructions for mRNA-Seq sample preparation (Illumina, Inc., San Diego, CA, USA). The cDNA library products were sequenced by Illumina paired-end sequencing technology with read lengths of 100 bp, and they were sequenced on an Illumina HiSeq 2500 instrument by Biomarker Technologies Co. Ltd. (Beijing, China) and BGI Tech Solutions Co., Ltd. (Shenzhen, China). The dataset were submitted to the NIH Short Read Archive (accession number: SRP062984).

### *De novo* Assembly

Reads from the eight libraries were assembled separately. All sequences smaller than 200 bp were eliminated because the overlap setting used for the assembly was 101 + 97 and 94 + 96 using paired-end sequencing. Trinity (http://trinityrnaseq.sourceforge.net/) consists of three software modules, Inchworm, Chrysalis, and Butterfly, which were applied sequentially to process large volumes of RNA-Seq reads.

### Unigene Annotation

Unigenes were annotated on the basis of a set of sequential BLAST searches (Altschul et al., 1997) performed with the aim to detect the most descriptive annotation for each sequence. The assembled sequences were compared with sequences in the National Center for Biotechnology Information (NCBI) non-redundant (Nr) protein and nucleotide (Nt) databases, the Swiss-Prot protein database, the Kyoto Encyclopedia of Genes and Genomes (KEGG) pathway database, and the Cluster of Orthologous Groups (COG) database. The unigenes were also annotated by the Blast2GO program (Conesa et al., 2005) and Gene Ontology (GO) functional classification was performed using WEGO software.

### Analysis of Differentially Expressed Genes (DEGs)

Gene expression levels were measured according to reads per kilobase per million reads (RPKM) in the RNA-Seq analyses (Mortazavi et al., 2008). IDEG 6 software (Romualdi et al., 2003) was used to identify DEGs by pairwise comparison, with an absolute value of log2 Ratio ≥ 1 and a FDR significance score <0.001.

## Results

### Phenotypic Analysis

**FIGURE 1:**
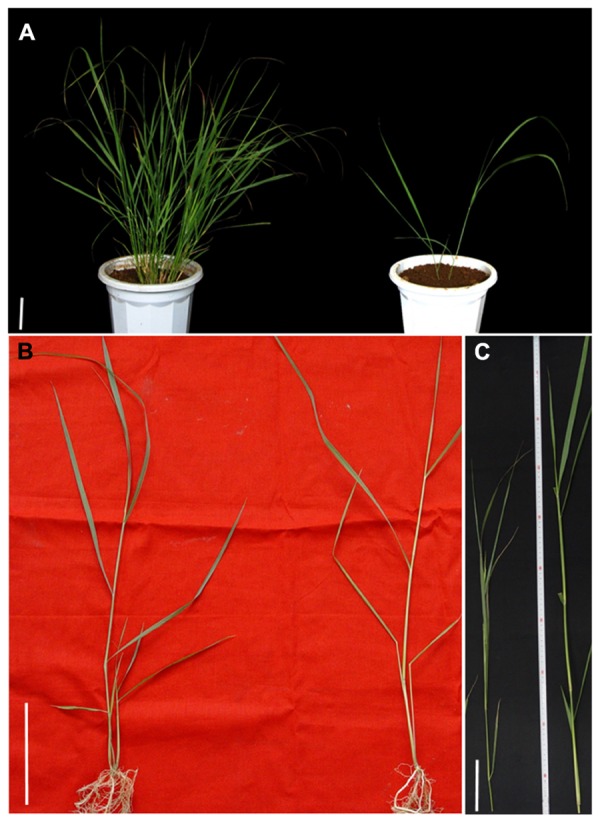
**Phenotype of switchgrass mutants *ht* and *lt.* (A)** Phenotype of two mutants at the first generation after mutagenesis. **(B)** Young seedling of *ht* and *lt* obtained by asexual propagation. **(C)** Tillers of *ht* and *lt* at heading stage. Left: *ht*, Right: *lt*. Bars = 10 cm.

The number of tillers in the *ht* (high tillering) switchgrass mutant was 36 before the first cutting and increased rapidly to 115 after the third cutting. However, in the *lt* (low tillering) group, the number was only two before the first cutting (**Figure 1A**) and seven after the third cutting. The asexually propagated *ht* mutant still displayed higher tillering ability than *lt* at the tillering stage (**Figure 1B**). The tiller size also differed significantly between the mutants (**Figure 1C**). Tiller height in *ht* and *lt* was 58.3 and 139.3 cm, respectively; tiller diameter was 2.2 and 3.8 cm, respectively. In addition, there were more roots in *ht* than *lt*, but the roots were thinner (**Figure 1B**). This result indicated that the root branching was different in the two mutants. In longitudinal sections of the shoot apex, there was no difference in the initiation of tiller buds in two mutants (Supplementary Figure S1). These results suggest that the change in tiller number in the two mutants was caused by alteration of tiller bud outgrowth and tiller elongation.

### Illumina Sequencing and *de novo* Assembly

To achieve an overview of the switchgrass tillering transcriptome and gene activity at Nt-level resolution, cDNA samples from tiller buds of the two plants were sequenced using the Illumina Genome Analyzer. To accurately determine the expression level of DEGs in the two mutants, L-1 and L-2 were selected from *lt* as duplications at the same generation, and H-1 and H-2 were selected from *ht* as the contrast. L-3 and L-4 and H-3 and H-4 were from the two mutants at another generation. Using stringent quality assessment and data filtering, the eight sequenced samples produced 101-bp independent reads from either end of a cDNA fragment. The amount of data from the eight samples exceeded 34G and the GC-rich content of the eight samples was approximately 53%. High-quality reads (with 100% Q20 base) were selected for further study. The eight sequences are summarized in **Table 1**.

**Table 1:** Output statistics of sequencing.

Sample	Total clean nucleotides (nt)	Q20 (%)	GC (%)
h-1	3,942,743,645	98.75	55.17
h-2	4,118,653,278	98.81	54.15
h-3	4,691,323,440	96.99	52.20
h-4	4,464,934,560	96.90	52.93
l-1	4,338,797,093	98.69	54.07
l-2	3,843,755,733	98.75	52.21
l-3	4,606,409,880	97.00	52.24
l-4	4,626,284,580	96.96	51.90

**FIGURE 2:**
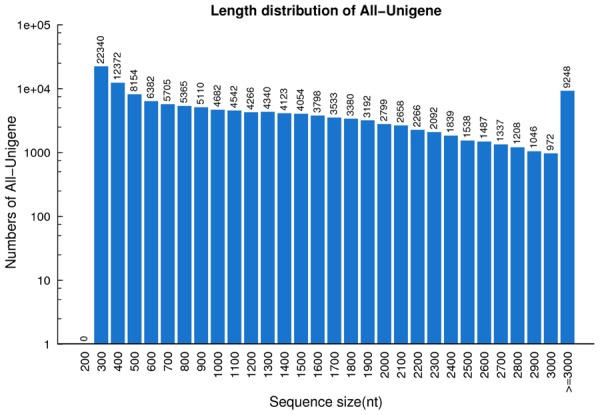
**Length distribution of unigenes in switchgrass**. The horizontal coordinates are unigene lengths and the vertical coordinates are numbers of unigenes.

Utilizing the Trinity *de novo* assembly program (Grabherr et al., 2011), 133,828 unigenes were detected. The total length for unigenes was 165,666,331 nt, with an average length of 1,238 nt and N50 of 1,890 nt. In total 38,027 transcripts (28.41%) had a length of 1–2 kb, 16,443 transcripts (12.29%) had a length of 2–3 kb, and 9,248 transcripts (6.91%) were longer than 3 kb. Approximately half of the unigenes (63,918) were >1 kb (**Figure 2**). These results indicate that high-quality transcriptome data were captured in this study. These datasets will provide a beneficial contribution to the transcriptome of switchgrass (Wang et al., 2012; Wilson et al., 2013; Xie et al., 2014).

### Annotation of all Assembled Unigenes

**FIGURE 3:**
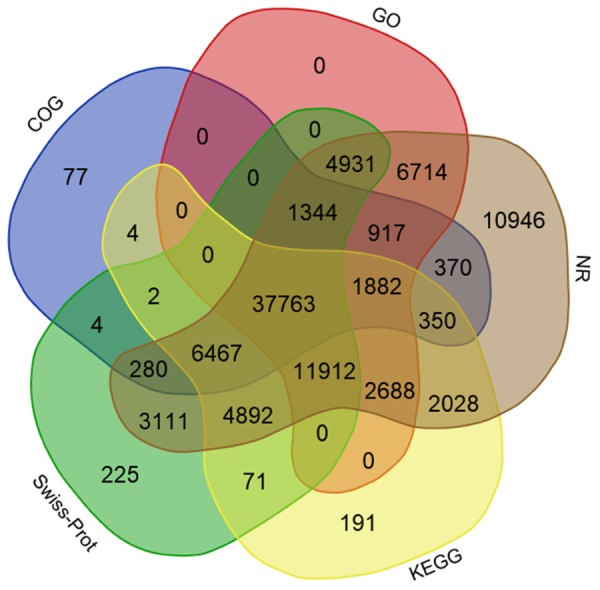
**Distribution of unigenes annotated in different databases**.

Using several complementary approaches, we annotated the unigenes in diverse protein databases including the NCBI Nr protein database, UniProt/Swiss-Prot, KEGG, COGs of proteins (COG), and GO. Based on an *E*-value lower than 10^-5^, the best match was chosen (**Table 2**). First, utilizing the BLAST (Basic Local Alignment Search Tool) algorithm, a sequence similarity search was operated against the above databases. In total, 96,595 unigenes were significantly been matched in the Nr database and 71,002 unigenes were annotated in the Swiss-Prot database. Moreover, 68,250 unigenes matched in KEGG annotation, 49,460 unigenes matched in COG annotation, and 68,151 unigenes matched in GO annotation. However, 24,681 unigenes did not match in the databases evaluated. The distribution and overlaps of unigenes from different databases are displayed in **Figure 3**. In total, 37,763 unigenes matched in all of the databases.

**Table 2:** Summary statistics for functional annotation in a different database.

Annotated database	Unigenes	Percentage (%)
Non-redundant (NR)	96,595	72.18
Swiss-Prot	71,002	53.05
Kyoto Encyclopedia of Genes and Genome (KEGG)	68,250	51.00
Cluster of Orthologous Group (COG)	49,460	36.96
Gene Ontology (GO)	68,151	50.92

**FIGURE 4:**
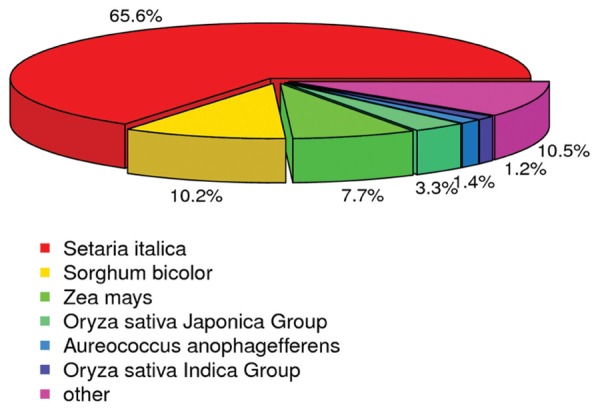
**Species distribution of the result of non-redundant (NR) annotation**.

Annotation was also analyzed to estimate the gene proportions of other plants represented in the switchgrass transcriptome data. According to the Nr annotation database, 63,335 (65.6%) unigenes were most closely related to genes of *Setaria italica*, 9,852 (10.20%) unigenes were most closely related to genes of *Sorghum bicolor*, and 7,478 (7.74%) unigenes were most closely to genes of *Zea mays* (**Figure 4**). These results indicated that millet was the best match among the translated switchgrass contig sequences, followed by sorghum and maize. Therefore, although switchgrass is polyploid, its gene space is not different from that of other grass (Wang et al., 2012).

### Analysis of Differentially Expressed Genes

To further study the tillering information, DEGs were detected using general chi squared analysis in IDE6 software and the assay was adjusted using the FDR (false discovery rate; <0.001). Genes in the two mutants in which the highest expression was more than two times the lowest expression were considered to be differentially expressed.

**FIGURE 5:**
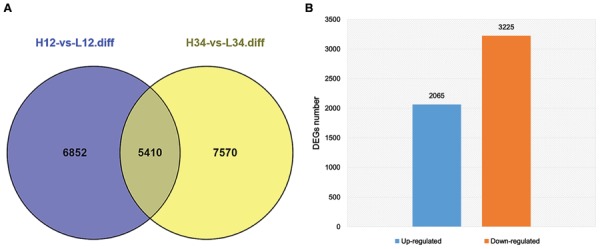
**Concordant Differentially expressed genes (DEGs) of *ht* and *lt* from two generations. (A)** DEGs of H-1 and H-2, and L-1 and L-2 are indicated in blue; those of H-3 and H-4, and L-3 and L-4 are indicated in pink. The overlap region indicates the number of common DEGs in the two groups. **(B)** The number of concordant up-regulated and down-regulated DEGs excluding unigenes with contrary variation trend in the two groups.

Tillering with monocots is a well-known agronomic trait that involves a complicated development process. Although many genes and their mechanisms of regulating tillering have been extensively studied in plants, the mechanisms underlying tillering remain to be elucidated. In this study, DEGs were identified from H1 and H2 and L1 and L2 at the same generation (FDR < 0.001, Log2ratio > 1). Other DEG data were from H3 and H4, and L3 and L4 of another generation. Thus, 5,410 DEGs from the two experimental groups were detected (**Figure 5A**). In total, 5,290 DEGs, including 3,225 up-regulated DEGs and 2,065 down-regulated DEGs, were selected for subsequent study, excluding the unigenes with a contrary variation trend in the two groups (**Figure 5B**). For 2,526 (47.75%) unigenes, including 1,279 up-regulated and 1,247 down-regulated genes, the log_2_ (L/H) was 1–3, and differential expression was thus not observed in these two mutants. In contrast, in 839 unigenes (104 up-regulated and 735 down-regulated), the log_2_ (L/H) exceeded 10, and these genes were thus significantly up-regulated or down-regulated in the two mutants (**Table 3**). These differentially expressed unigenes underlie the difference in tillering ability in the two mutants.

**Table 3:** Expression statistics of differentially expressed genes (DEGs).

Log_2_ratio (L/H)	Total DEGs	DEG number	
		Up	Down
1–3	2,526	1,279	1,247
3–6	1,360	489	871
6–10	565	193	372
>10	839	104	735

### GO Functional Classification of DEGs and Unigenes

**FIGURE 6:**
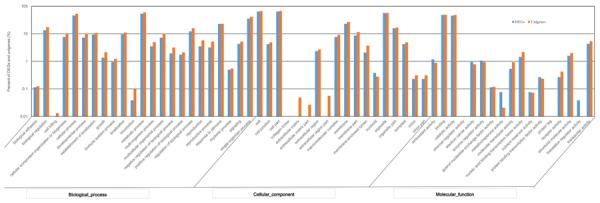
**Gene Ontology (GO) functional classification of DEGs and total unigenes in switchgrass tillering mutants**.

Using the GO annotation, 2,260 DEGs were assigned to 50 subcategories. Among them, 1,752 DEGs were involved in biological processes, 1,781 DEGs were related to cellular components, and 1,875 DEGs were grouped under molecular functions. Within the biological process category, the most represented DEGs classified as “metabolic process,” “cellular process,” and “single-organism process.” Within the cellular component category, the most represented DEGs were classified as “cell,” “cell part,” and “organelle.” Within the molecular function category, the largest proportion of DEGs was classified as “binding,” “catalytic activity” and “transporter activity” (**Figure 6**). Compared to the GO classification of DEGs and all unigenes, the proportion of unigenes from DEGs involved in “electron carrier activity,” “antioxidant activity,” and “enzyme regulator activity” was larger than that of all unigenes, indicating that these DEGs may be involved in tillering.

### COG Functional Classification of DEGs and Unigenes

**FIGURE 7:**
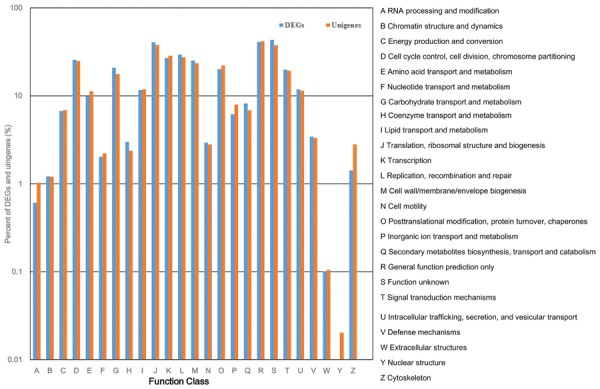
**Cluster of Orthologous Group (COG) classification of DEGs and total unigenes in switchgrass tillering mutants**.

All of the DEGs were submitted to the COG database to search for functional prediction and classification, and 1,913 unigenes were assigned to 25 COG functional categories. Among these categories, the cluster of “function unknown” was the largest group, followed by “general function prediction”; “translation, ribosomal structure, and biogenesis”; “replication, recombination, and repair”; “transcription”; and “cell cycle control, cell division, and chromosome partitioning.” The smallest groups were “chromatin structure and dynamics,” “RNA processing and modification,” and “extracellular structures” (**Figure 7**). The percentage of unigenes involved in “coenzyme transport and metabolism”; “secondary metabolites biosynthesis, transport, and catabolism”; and “carbohydrate transport and metabolism” was higher in DEGs than in all unigenes considered together, indicating that these three functional categories are important for tiller development.

### KEGG Pathway Mapping of DEGs

Functional classification and pathway assignment of DEGs was performed using KEGG (Kanehisa et al., 2004). In total, 2,955 DEGs among all DEGs were mapped to 122 KEGG pathways using blastx with an E-value cutoff of <1 × 10^-5^. The pathways containing the most DEGs were involved in “metabolic pathways” (907 unigenes), followed by “RNA transport” (697 unigenes), “mRNA surveillance pathway” (641 unigenes), “plant–pathogen interaction” (348 unigenes), “glycerophospholipid metabolism” (328 unigenes), “endocytosis” (326 unigenes), “biosynthesis of secondary metabolites” (322 unigenes), and “ether lipid metabolism” (311 unigenes). The results are summarized in Supplementary Table S1. Comparison with the distribution of all unigenes in KEGG pathways revealed enrichment of DEGs in pathways such as “pyrimidine metabolism,” “homologous recombination,” “zeatin biosynthesis,” “fatty acid metabolism,” “photosynthesis,” “propanoate metabolism,” “valine, leucine and isoleucine degradation,” “flavone and flavonol biosynthesis,” and “sesquiterpenoid and “triterpenoid biosynthesis” (**Table 4**). Of these pathways, “zeation biosynthesis,” “flavone and flavonol biosynthesis,” and “triterpenoid biosynthesis” were related to synthesis of plant hormones. These results indicate that DEGs involved in these pathways may play an important role in tillering of switchgrass.

**Table 4:** Differentially expressed genes with significantly enriched pathways.

Pathways	DEGs genes with pathway annotation (2955)	All genes with pathway annotation (68250)
mRNA surveillance pathway	641 (21.69%)	12,117 (17.75%)
Plant–pathogen interaction	348 (11.78%)	4,070 (5.96%)
Glycerophospholipid metabolism	328 (11.1%)	6,354 (9.31%)
Endocytosis	326 (11.03%)	6,431 (9.42%)
Purine metabolism	169 (5.72%)	2,083 (3.05%)
Pyrimidine metabolism	163 (5.52%)	1,966 (2.88%)
Homologous recombination	47 (1.59%)	604 (0.88%)
Zeatin biosynthesis	31 (1.05%)	362 (0.53%)
Flavonoid biosynthesis	31 (1.05%)	523 (0.77%)
Fatty acid metabolism	28 (0.95%)	323 (0.47%)
Stilbenoid, diarylheptanoid, and gingerol biosynthesis	27 (0.91%)	429 (0.63%)
Tyrosine metabolism	27 (0.91%)	433 (0.63%)
Terpenoid backbone biosynthesis	24 (0.81%)	396 (0.58%)
Benzoxazinoid biosynthesis	20 (0.68%)	177 (0.26%)
Photosynthesis	20 (0.68%)	214 (0.31%)
Propanoate metabolism	19 (0.64%)	182 (0.27%)
Flavone and flavonol biosynthesis	19 (0.64%)	244 (0.36%)
Cutin, submarine and wax biosynthesis	15 (0.51%)	233 (0.34%)
Valine, leucine, and isoleucine degradation	13 (0.44%)	275 (0.4%)
Sesquiterpenoid and triterpenoid biosynthesis	5 (0.17%)	55 (0.08%)

## Discussion

In this work, two tillering mutants (*ht* and *lt*) that displayed high and low tillering were generated by EMS mutagenesis. The phenotype was caused by disrupted bud outgrowth, as the tiller bud primordium development was unchanged. The tiller outgrowth was regulated in a complicated manner by genetic and environmental factors. The tillering mutants in this study must be propagated by asexual reproduction, as switchgrass is allogamous and strongly incompatible with selfing. The tillering ability of the two mutants was confirmed by cutting every generation and separating the tillers. To identify the mechanism of tiller bud outgrowth in switchgrass, young tiller buds were separated from the original mutants at two generations by cutting to extract RNA for sequencing. The other physiological properties of the two mutants remain to be discovered.

*De novo* transcriptome sequencing of RNA from *P. virgatum* buds was performed. Approximately 96,595 unigenes were significantly matched in the Nr database and 68,151 unigenes were matched in the GO annotation, whereas 49,460 unigenes were matched in the COG annotation and 68,250 unigenes were matched in the KEGG annotation. However, 24,681 unigenes were not matched in the present databases. The non-matched unigenes were aligned to the present switchgrass genome, and only approximately 19% were not aligned to the database. The possible reasons for the non-matched unigenes include the following. First, short reads obtained from sequencing would rarely be matched to known genes because the significance of the BLAST comparison depends in part on the length of the query sequence (Novaes et al., 2008; Liu et al., 2012a). Second, misassembly of the short reads may cause the sequence to be determined as non-existent in switchgrass. Third, the annotation of some of the unigenes may actually represent be non-coding RNAs. The insufficient amount of sequences of switchgrass in public databases also influences the annotation efficiency (Hou et al., 2011). Finally, the unmatched unigenes could be new genes that had never been discovered and annotated.

Approximately 5,290 differentially expressed unigenes were identified in two tillering mutants with detailed screening. These DEGs were primarily involved in metabolic pathways, RNA transport, mRNA surveillance, plant-pathogen interactions, glycerophospholipid metabolism, endocytosis, biosynthesis of secondary metabolites, and ether lipid metabolism. The results reflected the complicated regulation of tillering in switchgrass. DEGs with unknown function were the largest group in the COG functional analysis, indicating that the genes involved in tillering in switchgrass are complicated and require further study. Some DEGs were enriched in KEEG pathways predicted to contribute to plant hormone synthesis. Some unigenes that showed dramatic changes in expression between the two mutants were annotated in pathways of plant hormones regulating plant growth and development. All of these results indicate that the two tillering mutants of switchgrass were regulated in a complicated manner by plant hormones. The hormone content and responses to different hormones in the two mutants will be determined in the future.

Some genes listed in the introduction of this paper (*MOC1, TB1, PIN1, D3, HTD1/D17, D10, D14, D27, miR156, RFL, and MOC3/TBA1*) were found to regulate tillering in other species. Alignment was performed between DEGs of the two mutants and these referenced genes. A gene homologous to *D10* was found to be up-regulated in the low-tillering mutant, whereas homologs of *miR156* and *OsTB1* were down-regulated. These results indicate that similar pathways regulate tillering in switchgrass and rice, and the change in tillering in the two mutants may involve the strigolactone pathway. However, the expression of the *OsTB1* homolog was lower in low-tillering plants than in high-tillering plants. Therefore, other regulation pathways for tillering may exist.

In a previous study, two switchgrass inbred lines with contrasting tillering performance were selected for transcriptome profiling using Affymetrix gene chips (Wang et al., 2013). In total, 750 genes were up-regulated in the high-tillering inbred and 390 genes were up-regulated in the low-tillering inbred. A comparison of the findings of Wang et al. (2013) with those of the present study reveals some common genes with same trend in different tillering plants, including 135 unigenes are up-regulated in the high-tillering plants and 17 unigenes are up-regulated in the low-tillering plants. The common genes in the two studies deserved further study because they may play very important roles in regulating tillering in switchgrass. However, most of the DEGs were different between the two studies. The lack of agreement between the two studies may involve the different backgrounds of the switchgrass. Additionally, the developmental stages at which tillering differentiation occurred in the high-tillering and low-tillering plants may have been different between the two studies.

## Author Contributions

Conceived and designed the experiments: YX, FS. Performed the experiments: KX, FS, YW, GC. Analyzed the data: KX, FS, YW, LS. Wrote the paper: KX, FS, YX.

## Conflict of Interest Statement

The authors declare that the research was conducted in the absence of any commercial or financial relationships that could be construed as a potential conflict of interest.
