# Supplementary material for: De novo assembly and transcriptome analysis of two contrary tillering mutants to learn the mechanisms of tillers outgrowth in switchgrass (Panicum virgatum L.)
Source: Front Plant Sci. 2015 Sep 16;6:749. doi: 10.3389/fpls.2015.00749 (PMC4584987; doi:10.3389/fpls.2015.00749)
Supplement: Supplementary file 1 [file Data_Sheet_1.DOCX]

# *Supplementary Material*

# *De novo* assembly and transcriptome analysis of two contrary tillering mutants to learn the mechanisms of tillers outgrowth in switchgrass (*Panicum virgatum* L.)

**Kaijie Xu^†1 2^, Fengli Sun^†1^, Guaiqiang Chai^1^, Yongfeng Wang^1^,** **Lili Shi****^3^, Shudong Liu^1^, Yajun Xi^1^***

^1^ State Key Laboratory of Crop Stress Biology for Arid Areas, Northwest A&F University, Yangling, Shaanxi 712100, China.

^2^ Institute of Cotton Research of CAAS, Anyang, Henan, 455000, China.

^3^ HanDanShi Agriculture Academy of Sciences, Handan, Hebei, 056001, China.

**^†^** These authors contribute equally to this work.

* Correspondence: Yajun Xi, State Key Laboratory of Crop Stress Biology for Arid Areas, Northwest A&F University, Yangling, Shaanxi 712100, China.

[xiyajun11@126.com](mailto:xiyajun11@126.com).

1. **Supplementary figures and tables**

## Supplementary Tables

**Table S1. KEGG pathways of DEGs and all unigenes involved.**

| Pathway | DEGs genes with pathway annotation (2955) | All genes with pathway annotation (68250) | Pathway ID |
| --- | --- | --- | --- |
| Metabolic pathways | 907 (30.69%) | 18660 (27.34%) | ko01100 |
| RNA transport | 697 (23.59%) | 13799 (20.22%) | ko03013 |
| mRNA surveillance pathway | 641 (21.69%) | 12117 (17.75%) | ko03015 |
| Plant-pathogen interaction | 348 (11.78%) | 4070 (5.96%) | ko04626 |
| Glycerophospholipid metabolism | 328 (11.1%) | 6354 (9.31%) | ko00564 |
| Endocytosis | 326 (11.03%) | 6431 (9.42%) | ko04144 |
| Biosynthesis of secondary metabolites | 322 (10.9%) | 6839 (10.02%) | ko01110 |
| Ether lipid metabolism | 311 (10.52%) | 5952 (8.72%) | ko00565 |
| Purine metabolism | 169 (5.72%) | 2083 (3.05%) | ko00230 |
| Pyrimidine metabolism | 163 (5.52%) | 1966 (2.88%) | ko00240 |
| RNA polymerase | 144 (4.87%) | 1484 (2.17%) | ko03020 |
| Spliceosome | 127 (4.3%) | 3331 (4.88%) | ko03040 |
| Starch and sucrose metabolism | 88 (2.98%) | 2295 (3.36%) | ko00500 |
| RNA degradation | 86 (2.91%) | 1471 (2.16%) | ko03018 |
| Plant hormone signal transduction | 80 (2.71%) | 2680 (3.93%) | ko04075 |
| Pentose and glucuronate interconversions | 76 (2.57%) | 1335 (1.96%) | ko00040 |
| Protein processing in endoplasmic reticulum | 62 (2.1%) | 1933 (2.83%) | ko04141 |
| Ribosome biogenesis in eukaryotes | 61 (2.06%) | 1130 (1.66%) | ko03008 |
| Glycolysis / Gluconeogenesis | 59 (2%) | 964 (1.41%) | ko00010 |
| Homologous recombination | 47 (1.59%) | 604 (0.88%) | ko03440 |
| Phenylpropanoid biosynthesis | 39 (1.32%) | 1123 (1.65%) | ko00940 |
| Ribosome | 37 (1.25%) | 1131 (1.66%) | ko03010 |
| Peroxisome | 34 (1.15%) | 600 (0.88%) | ko04146 |
| Nucleotide excision repair | 34 (1.15%) | 725 (1.06%) | ko03420 |
| Zeatin biosynthesis | 31 (1.05%) | 362 (0.53%) | ko00908 |
| Flavonoid biosynthesis | 31 (1.05%) | 523 (0.77%) | ko00941 |
| Galactose metabolism | 31 (1.05%) | 664 (0.97%) | ko00052 |
| Fatty acid metabolism | 28 (0.95%) | 323 (0.47%) | ko00071 |
| Glycerolipid metabolism | 28 (0.95%) | 487 (0.71%) | ko00561 |
| Stilbenoid, diarylheptanoid and gingerol biosynthesis | 27 (0.91%) | 429 (0.63%) | ko00945 |
| Tyrosine metabolism | 27 (0.91%) | 433 (0.63%) | ko00350 |
| Amino sugar and nucleotide sugar metabolism | 27 (0.91%) | 853 (1.25%) | ko00520 |
| Pyruvate metabolism | 26 (0.88%) | 418 (0.61%) | ko00620 |
| DNA replication | 25 (0.85%) | 451 (0.66%) | ko03030 |
| Fructose and mannose metabolism | 25 (0.85%) | 582 (0.85%) | ko00051 |
| Terpenoid backbone biosynthesis | 24 (0.81%) | 396 (0.58%) | ko00900 |
| Circadian rhythm - plant | 24 (0.81%) | 511 (0.75%) | ko04712 |
| Pentose phosphate pathway | 24 (0.81%) | 525 (0.77%) | ko00030 |
| Oxidative phosphorylation | 24 (0.81%) | 727 (1.07%) | ko00190 |
| Ubiquitin mediated proteolysis | 24 (0.81%) | 976 (1.43%) | ko04120 |
| alpha-Linolenic acid metabolism | 23 (0.78%) | 299 (0.44%) | ko00592 |
| Glutathione metabolism | 22 (0.74%) | 474 (0.69%) | ko00480 |
| Cyanoamino acid metabolism | 22 (0.74%) | 553 (0.81%) | ko00460 |
| Cysteine and methionine metabolism | 21 (0.71%) | 642 (0.94%) | ko00270 |
| Benzoxazinoid biosynthesis | 20 (0.68%) | 177 (0.26%) | ko00402 |
| Photosynthesis | 20 (0.68%) | 214 (0.31%) | ko00195 |
| Propanoate metabolism | 19 (0.64%) | 182 (0.27%) | ko00640 |
| Flavone and flavonol biosynthesis | 19 (0.64%) | 244 (0.36%) | ko00944 |
| Other glycan degradation | 18 (0.61%) | 365 (0.53%) | ko00511 |
| Phenylalanine metabolism | 18 (0.61%) | 578 (0.85%) | ko00360 |
| ABC transporters | 18 (0.61%) | 646 (0.95%) | ko02010 |
| Limonene and pinene degradation | 17 (0.58%) | 309 (0.45%) | ko00903 |
| Mismatch repair | 17 (0.58%) | 397 (0.58%) | ko03430 |
| Phagosome | 17 (0.58%) | 645 (0.95%) | ko04145 |
| Carotenoid biosynthesis | 16 (0.54%) | 437 (0.64%) | ko00906 |
| Phosphatidylinositol signaling system | 16 (0.54%) | 455 (0.67%) | ko04070 |
| Cutin, suberine and wax biosynthesis | 15 (0.51%) | 233 (0.34%) | ko00073 |
| Protein export | 15 (0.51%) | 273 (0.4%) | ko03060 |
| Glycine, serine and threonine metabolism | 15 (0.51%) | 326 (0.48%) | ko00260 |
| Basal transcription factors | 15 (0.51%) | 475 (0.7%) | ko03022 |
| Diterpenoid biosynthesis | 14 (0.47%) | 212 (0.31%) | ko00904 |
| Tryptophan metabolism | 14 (0.47%) | 219 (0.32%) | ko00380 |
| Phenylalanine, tyrosine and tryptophan biosynthesis | 14 (0.47%) | 281 (0.41%) | ko00400 |
| Base excision repair | 14 (0.47%) | 306 (0.45%) | ko03410 |
| Arginine and proline metabolism | 14 (0.47%) | 433 (0.63%) | ko00330 |
| Riboflavin metabolism | 13 (0.44%) | 203 (0.3%) | ko00740 |
| Isoflavonoid biosynthesis | 13 (0.44%) | 226 (0.33%) | ko00943 |
| Ubiquinone and other terpenoid-quinone biosynthesis | 13 (0.44%) | 232 (0.34%) | ko00130 |
| Valine, leucine and isoleucine degradation | 13 (0.44%) | 275 (0.4%) | ko00280 |
| Ascorbate and aldarate metabolism | 13 (0.44%) | 320 (0.47%) | ko00053 |
| Citrate cycle (TCA cycle) | 13 (0.44%) | 349 (0.51%) | ko00020 |
| Biosynthesis of unsaturated fatty acids | 12 (0.41%) | 215 (0.32%) | ko01040 |
| Fatty acid elongation | 12 (0.41%) | 222 (0.33%) | ko00062 |
| Aminoacyl-tRNA biosynthesis | 12 (0.41%) | 335 (0.49%) | ko00970 |
| Steroid biosynthesis | 11 (0.37%) | 310 (0.45%) | ko00100 |
| Carbon fixation in photosynthetic organisms | 11 (0.37%) | 322 (0.47%) | ko00710 |
| Glyoxylate and dicarboxylate metabolism | 11 (0.37%) | 403 (0.59%) | ko00630 |
| Inositol phosphate metabolism | 10 (0.34%) | 357 (0.52%) | ko00562 |
| Lysine degradation | 9 (0.3%) | 219 (0.32%) | ko00310 |
| Sphingolipid metabolism | 9 (0.3%) | 244 (0.36%) | ko00600 |
| Regulation of autophagy | 9 (0.3%) | 317 (0.46%) | ko04140 |
| beta-Alanine metabolism | 8 (0.27%) | 178 (0.26%) | ko00410 |
| Glycosylphosphatidylinositol(GPI)-anchor biosynthesis | 8 (0.27%) | 238 (0.35%) | ko00563 |
| Natural killer cell mediated cytotoxicity | 8 (0.27%) | 275 (0.4%) | ko04650 |
| Non-homologous end-joining | 7 (0.24%) | 72 (0.11%) | ko03450 |
| Alanine, aspartate and glutamate metabolism | 7 (0.24%) | 241 (0.35%) | ko00250 |
| Vitamin B6 metabolism | 6 (0.2%) | 92 (0.13%) | ko00750 |
| Glycosaminoglycan degradation | 6 (0.2%) | 130 (0.19%) | ko00531 |
| N-Glycan biosynthesis | 6 (0.2%) | 193 (0.28%) | ko00510 |
| SNARE interactions in vesicular transport | 6 (0.2%) | 220 (0.32%) | ko04130 |
| Sesquiterpenoid and triterpenoid biosynthesis | 5 (0.17%) | 55 (0.08%) | ko00909 |
| Valine, leucine and isoleucine biosynthesis | 5 (0.17%) | 98 (0.14%) | ko00290 |
| Histidine metabolism | 5 (0.17%) | 100 (0.15%) | ko00340 |
| Tropane, piperidine and pyridine alkaloid biosynthesis | 5 (0.17%) | 169 (0.25%) | ko00960 |
| Isoquinoline alkaloid biosynthesis | 5 (0.17%) | 176 (0.26%) | ko00950 |
| Monoterpenoid biosynthesis | 4 (0.14%) | 73 (0.11%) | ko00902 |
| Photosynthesis - antenna proteins | 4 (0.14%) | 74 (0.11%) | ko00196 |
| Glycosphingolipid biosynthesis - globo series | 4 (0.14%) | 74 (0.11%) | ko00603 |
| Nicotinate and nicotinamide metabolism | 4 (0.14%) | 76 (0.11%) | ko00760 |
| Sulfur metabolism | 4 (0.14%) | 116 (0.17%) | ko00920 |
| Pantothenate and CoA biosynthesis | 4 (0.14%) | 119 (0.17%) | ko00770 |
| Circadian rhythm - mammal | 4 (0.14%) | 124 (0.18%) | ko04710 |
| Brassinosteroid biosynthesis | 4 (0.14%) | 152 (0.22%) | ko00905 |
| Fatty acid biosynthesis | 4 (0.14%) | 180 (0.26%) | ko00061 |
| Porphyrin and chlorophyll metabolism | 4 (0.14%) | 229 (0.34%) | ko00860 |
| Glucosinolate biosynthesis | 3 (0.1%) | 48 (0.07%) | ko00966 |
| Thiamine metabolism | 3 (0.1%) | 51 (0.07%) | ko00730 |
| Glycosphingolipid biosynthesis - ganglio series | 3 (0.1%) | 64 (0.09%) | ko00604 |
| Arachidonic acid metabolism | 3 (0.1%) | 73 (0.11%) | ko00590 |
| Folate biosynthesis | 3 (0.1%) | 95 (0.14%) | ko00790 |
| Lysine biosynthesis | 3 (0.1%) | 108 (0.16%) | ko00300 |
| Butanoate metabolism | 3 (0.1%) | 199 (0.29%) | ko00650 |
| Proteasome | 3 (0.1%) | 264 (0.39%) | ko03050 |
| Selenocompound metabolism | 2 (0.07%) | 54 (0.08%) | ko00450 |
| Sulfur relay system | 2 (0.07%) | 66 (0.1%) | ko04122 |
| One carbon pool by folate | 2 (0.07%) | 82 (0.12%) | ko00670 |
| Other types of O-glycan biosynthesis | 2 (0.07%) | 83 (0.12%) | ko00514 |
| Caffeine metabolism | 1 (0.03%) | 10 (0.01%) | ko00232 |
| Lipoic acid metabolism | 1 (0.03%) | 22 (0.03%) | ko00785 |
| Anthocyanin biosynthesis | 1 (0.03%) | 22 (0.03%) | ko00942 |
| Taurine and hypotaurine metabolism | 1 (0.03%) | 52 (0.08%) | ko00430 |
| Nitrogen metabolism | 1 (0.03%) | 185 (0.27%) | ko00910 |

## Supplementary figures


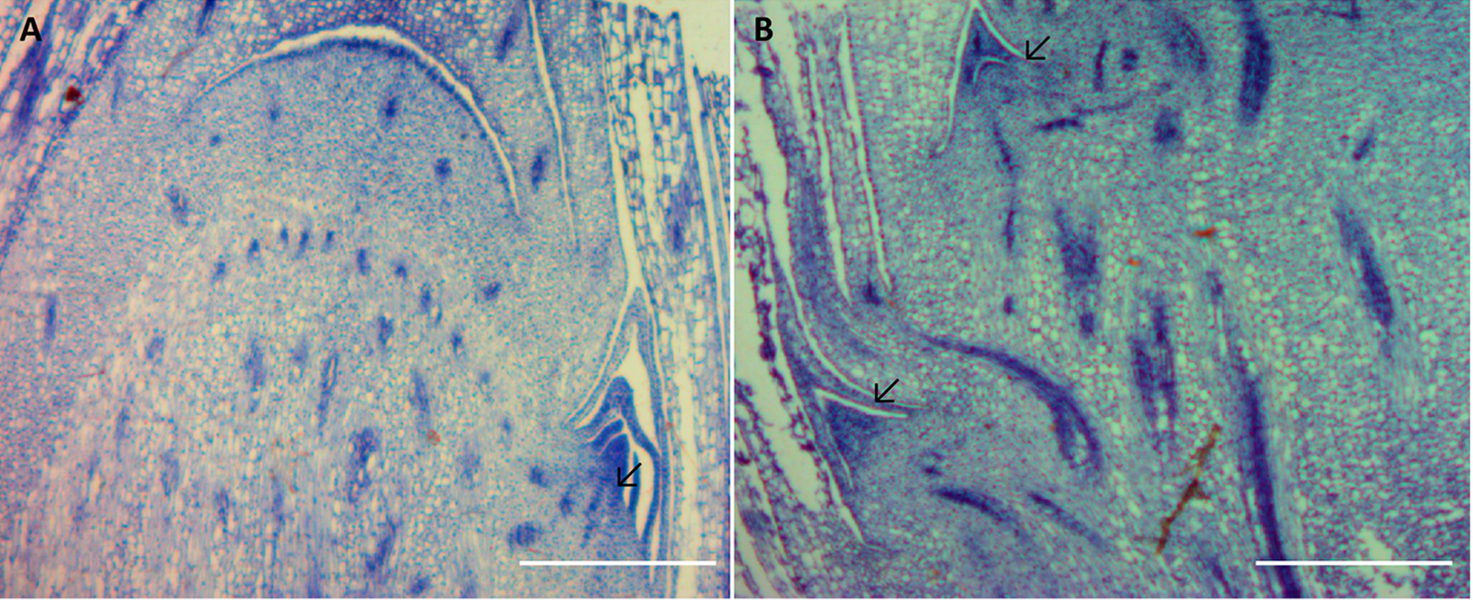


**Figure S1. The initiation of tiller bud in two mutants.** A: *ht* B: *lt.* There were no obvious difference between *ht* and *lt.* Bars=0.5 mm. Brackets indicated the buds.
